# Research Progress of Therapeutic Enzymes and Their Derivatives: Based on Herbal Medicinal Products in Rheumatoid Arthritis

**DOI:** 10.3389/fphar.2021.626342

**Published:** 2021-03-16

**Authors:** Ming Cai, Wei-Jian Ni, Lan Han, Wei-Dong Chen, Dai-Yin Peng

**Affiliations:** ^1^ Department of Pharmacy, The Second Affiliated Hospital of Anhui University of Chinese Medicine, Hefei, China; ^2^ Anhui University of Chinese Medicine, Hefei, China; ^3^ Key Laboratory of Chinese Medicinal Formula Research, Anhui University of Chinese Medicine, Hefei, China; ^4^ School of Pharmacy, Anhui Medical University, The Key Laboratory of Anti-inflammatory and Immune Medicines, Ministry of Education, Hefei, China; ^5^ Department of Pharmacy, Anhui Provincial Hospital, The First Affiliated Hospital of USTC, University of Science and Technology of China, Hefei, China

**Keywords:** rheumatoid arthritis, herbal medicinal products, inflammatory, immune, enzyme, enzyme derivatives

## Abstract

Rheumatoid arthritis (RA) acts as one of the most common, agnogenic and chronic inflammatory-autoimmune disorder which is characterized by persistent synovitis, cartilage destruction, and joint deformities, leads to a wide range of disabilities, and increased mortality, thus imposing enormous burdens. Several drugs with anti-inflammatory and immunomodulatory properties such as celecoxib, diclofenac and methotrexate are being selected as conventional drugs in the allopathic system of medicine for the treatment of RA in clinic. However, there are some serious side effects more or less when using these drugs because of their short poor bioavailability and biological half-life for a long time. These shortcomings greatly promote the exploration and application of new low- or no-toxicity drugs for treating the RA. Meanwhile, a growing number of studies demonstrate that several herbs present certain anti-inflammatory and anti-arthritic activities through different enzymes and their derivatives, which indicate that they are promising therapeutic strategies when targeting these mediators based on herbal medicinal products in RA research. This review article summarizes the roles of the main enzymes and their derivatives during the pathogenesis of RA, and clearly clarifies the explicit and potential targeted actions of herbal medicinal products that have anti-RA activity. Our review provides timely and critical reference for the scientific rationale use of herbal medicinal products, with the increasing basic research and clinical application of herbal medicinal products by patients with RA.

## Introduction

Rheumatoid arthritis (RA) is one of the most common agnogenic and chronic inflammatory-autoimmune disorder that major targets the synovium, joints, and cartilage, which causes irreversible joint damage, and causes severe extra-articular manifestations and complications (Fert-Bober et al., 2020). During the occurrence and development of rheumatoid arthritis, both environmental and genetic are important factors involved (Scherer et al., 2020). In the initiation phase of rheumatoid arthritis, there is the autoreactive T cells activation, the T cell mobilization and recruitment along with other leukocytes into the disease area, including joints, synovium, and cartilage (Fang et al., 2020). For the moment, these leukocytes produce multiple enzymes, various inflammatory cytokines and mediators such as phospholipase A_2_ (PLA_2_), prostaglandins (PG), and diverse cytokines (interleukins, tumor necrosis factor, etc), which induce the synovial and joint inflammation and finally cause the damage in joint tissue through triggering different signaling cascades intracellular and extracellular (McInnes and Schett, 2011; Hong et al., 2020). Therefore, targeting these enzymes and their derivatives is a potential therapeutic strategy for the treatment of rheumatoid arthritis. In recent years, using the well-defined biochemical and pharmacological inhibitors to suppress rheumatoid arthritis in experimental animal models have been reported many times (Croia et al., 2019). Interestingly, studies find that many of these enzymes, derivatives and related signaling pathways can be targeted and intervened by several medicinal products, for instance, herbal medicinal products which belong to the traditional medicine or complementary and alternative medicine (Mateen et al., 2016; Wang et al., 2020). Therefore, this review article summarizes the roles of main enzymes and their derivatives of inflammation during the occurrence and pathogenesis of RA, to provide insight into how the herbal medicinal products that target these enzymes and their derivatives may lead to the prevention of RA.

In this review, the herbal medicinal products we discuss below are examined through many experiments for their anti-inflammatory, anti-arthritic and immunoregulatory activities. Reviewing the related literature, we find that the *in vitro* researches are performed by those cultured defined cell types, including chondrocytes (Feng and Qiu, 2018), macrophages (McHugh, 2017; 2019), and fibroblasts (Croft et al., 2019), while the *in vivo* studies are based on multiple well-established experimental RA models, such as collagen-induced arthritis (CIA) (Kim et al., 2015; Li et al., 2017), adjuvant-induced arthritis (AIA) (Pan et al., 2017; Wang et al., 2017), as well as streptococcal cell wall-induced arthritis. In these studies, specific purified compounds, extractives and monomer derived from the herbal medicinal product are appended to the cultured cells in the case of inflammatory stimulants such as interleukin-1β (IL-1β), tumor necrosis factor-α (TNF-α) and lipopolysaccharide (LPS) for the *in vitro* experiments. The cells used for studies are taken from mice, rats or from the authoritative cell lines (Cheng et al., 2020; Yang et al., 2020). For the *in vivo* tests, the herbal medicinal products are investigated as an extract or a purified bioactive compound (Xiong et al., 2019). During the *in vivo* research, the intraperitoneal injection and oral administration are considered as the two-principal means of intervention (Bao et al., 2019). The parameters for assessing the RA model situation and the herbal medicinal products therapeutic efficacy are consisted of clinical criteria for grading such as phenotypic (weight change, paw volume), biochemical changes, histopathological analysis and RA biomarkers, et al.

## Therapeutic Enzymes and Their Derivatives in RA

As we know, RA is a common and agnogenic chronic inflammatory disorder. And meanwhile, inflammation is a kind of physiological stress reaction of the organism response to different external stimulus and internal anomaly such as infection, trauma, and immune reactions (Cronstein et al., 2020). During the initial period and perpetuation of inflammatory reaction, there are a variety of enzymes and their derivatives act in concert (Spel and Martinon, 2020). In this article, we discuss the detailed characteristics of these major enzymes and their derivatives including phospholipase A_2_ (PLA_2_), cyclooxygenase (COX) and prostaglandins (PGs), lipoxygenase (LOX), matrix metalloproteases (MMPs)/tissue inhibitors of metalloproteases (TIMPs), nitric oxide synthase (NOS) and nitric oxide (NO), as well as indoleamine 2, 3-dioxygenase (IDO), and demonstrate the targeting of these spots by natural herbal and synthetic products resulting in the prevention of RA (Table 1). Also, we try to clarify the mechanisms of these enzymes and their derivatives involved in the RA progression.

**TABLE 1:** List of herbal medicinal products involved in targeting the enzymes in rheumatoid arthritis.

Herbal products	Botanical source	Principle extracted	Study Phase	Subject	Target enzyme	Outcomes	References
PG201	12 herbs compound	Ethanol extract	Clinical study	Raw 264.7 macrophage cell; DBA/1 mice; Rabbit and OA patients	cPLA2	Relieve RA	Park et al. (2005), Yoo et al. (2014), and Kim et al. (2016)
Boerhaavia diffusa	Nyctaginaceae tuberous root	Ethanol extract	Preclinical study	Swiss albino mice; Swiss albino rat	sPLA2	Relieve RA	Giresha et al. (2017)
HMBA	Hemidesmus indicus R. BR	Ethanol extract	Preclinical study	New Zealand strain rabbit BALBc mice	sPLA2	Relieve RA	Gomes et al. (2012)
Danshen extract	Root of salvia miltiorrhiza bunge	80% ethanol extract	Clinical study	Human	sPLA2	Relieve RA	Chen et al. (2017)
TWHF extract	Tripterygium wilfordii hook F	Methanol extract; Chloroform extract	Preclinical study	Synovial fibroblast	COX-2	Relieve RA	Tao et al. (1998) and Lin et al. (2007)
Myricetin	Euphorbia dracunculoides	Ethanol + water extract	Preclinical study	BV2 cell Mice	COX-2	Relieve RA	Pan et al. (2019)
Xanthones extract	Swertia chirayita	Petroleum ether extract; Ethyl acetate extract	Preclinical study	Raw 264.7 macrophage cell	COX-2	Relieve RA	Hu et al. (2019)
Bacopa monniera extract	Bacopa monniera linn	Methanolic extract	Preclinical study	Wistar rat, Rat mononuclear cells	5-LOX/15-LOX	Relieve RA	Viji and Helen (2008)
HOEC	Incarvillea mairei var	80% ethanol extract	Preclinical study	Wistar rats	5-LOX/15-LOX	Relieve RA	Yang et al. (2018)
SOG	Saposhnikovia divaricata (turcz.) schischk	Ethyl acetate/n-butanol/ethanol/water (1:1:0.1:2, v/v/v/v)	Preclinical study	Raw 264.7 macrophage cell	5-LOX	Relieve RA	Zhao et al. (2016) and Liu et al. (2020)
TGP	Root of paeonia lactiflora pallas	Ethanol extract	Preclinical study	DBA/1 mice	MMP-1/MMP-3	Relieve RA	Li et al. (2019) and Can et al. (2020)
Celastrol	Root bark of tripterygium wilfordii hook. f	Ethylacetate extract	Preclinical study	Sprague-dawley rat	MMP-9	Relieve RA	Xiao et al. (2018)
Ikarisoside	Epimedium koreanum	Methanol extract	Preclinical study	Raw 264.7 macrophage cell	MMP-9	Relieve RA	Choi et al. (2010)
Ursolic acid	Ocimum sanctum	Methanol crude extract	Preclinical study	Albino wistar rat	MMP-2/MMP-9	Relieve RA	Radhiga et al. (2019)
Catechins	Green tea derived from the leaves of camellia sinensis	Acetone extract, Aqueous extract	Preclinical study	Human synovial fibroblast	MMP-2	Relieve RA	Fechtner et al. (2017)
POEa/POEe	Polygonum orientale L	Ethyl acetate extract; Ethyl ether extract	Preclinical study	Sprague-dawley rat	iNOS	Relieve RA	Gou et al. (2018)
Celastrus ethylacetate extract	Celastrus aculeatus merr	Ethylacetate extract	Preclinical study	Sprague-dawley rat	NOS	Relieve RA	Bai et al. (2014)
Celastrol	Root bark of *tripterygium wilfordii hook. f*	Ethylacetate extract	Preclinical study	Wistar rat	iNOS	Anti-inflammation	Kannaiyan et al. (2011)
Hemerocallis citrine, Ethanol extract	Hemerocallis citrine	Ethanol extract	Preclinical study	Wistar rat	IDO	Anti-inflammation	Liu et al. (2014)
Feiji recipe	12 herbs compound	Herbal mixture	Clinical study	C57BL/6 mice	IDO	Regulate immune	Luo et al. (2018)

Note: Preclinical: Research involving animals and cells; Clinical study: Research involving human specimens.

### Phospholipase A_2_ (PLA_2_)

PLA_2_ is a kind of intensively studied hydrolase which can catalyze-hydrolyze the membrane phospholipids at the position of sn-2 thereby producing the lysophospholipid and fatty acid products (Martin et al., 2020). Then, the produced free fatty acid (FFA) can be broken down into various importance biological lipid mediators, and the lysophospholipid products also play vital roles in the corresponding biological processes and physiological activities (Duchez et al., 2019). PLA_2_ is of high pharmaceutical value protein because it can be responsible for the arachidonic acid release from membranes, and subsequent transformation of fatty acid to leukotrienes and prostaglandins, which plays important roles in the subsequent inflammatory response (Magrioti and Kokotos, 2013). In organisms, there are more than 14 different species of PLA_2_ enzymes, which may play different or similar roles in many biological processes. Among these 14 isoforms, four main subtypes of PLA_2_ include the cytosolic phospholipase A_2_ (cPLA_2_), calcium-independent phospholipase A_2_ (iPLA_2_), secreted phospholipase A_2_ (sPLA_2_), and the platelet activating factor acetyl hydrolase/oxidized lipid lipoprotein-associated phospholipase A_2_ (LpPLA_2_) are widely studied and identified by researchers (Kozaki et al., 2015; Sodergren et al., 2015). cPLA_2_ is one of the main subtypes produced in the inflammation area and also the only one PLA_2_ with a catalyzed-hydrolysis peculiarity for arachidonic acid (AA) at the position of sn-2 in phospholipids (Hartz et al., 2019). As AA is the precursor of eicosanoids, so, cPLA_2_ can be considered as the pivotal enzyme involved in the production of eicosanoids and therefore, is an important enzyme in some inflammation diseases, e.g., RA (Sommerfelt et al., 2015). In addition to the above functions, cPLA_2_ can boost the enzymatic activity of nicotinamide adenine dinucleotide phosphate (NADPH) oxidase in monocytes and neutrophils to produce the superoxides during the process of inflammation (Raichel et al., 2008). Therefore, inhibiting the cPLA_2_ could simultaneously decrease the activities of multiple lipid materials, which facilitate the neutrophils recruitment to the inflammation area, and accelerate the superoxides production and release. Several studies show that cPLA_2_ is expressed in RA synovium (Malaviya et al., 2006), and has been performing significant roles during the progression of inflammatory in several models of arthritis (Courties et al., 2011). In the rheumatoid arthritis synovial fibroblasts (RASFs) of human, the expression level of cPLA_2_ is increased by proinflammatory cytokines such as TNF-α and IL-1β (Chi et al., 2011). Then, the elevated cPLA_2_ acts as an important regulator of those key players including interleukin-8 (IL-8), prostaglandin E2 (PGE_2_), stromelysin-1 (matrix metalloproteinase 3, MMP3) and COX2 in the pathology of RA, which results in the destruction, angiogenesis of bone and cartilage and the neutrophil recruitment (Sommerfelt et al., 2013). Common research has long held that the cartilage and bone degradation are the two major hallmarks of RA, therefore reduction or prevention of these destructive processes should be a central therapeutic objective. Also, in murine collagen induced arthritis, cPLA_2_ is also recognized to be one of the vital regulators of the neutrophil recruitment and inflammatory reaction, which highlights the promising biological relevance of cPLA_2_ in synovitis and arthritis (Raichel et al., 2008). Given all the evidence above, regulating and intervening the enzymatic activity of cPLA_2_ by specific cPLA_2_ inhibitors and then promoting the normalization of downstream signals probably be considered as a promising alternative or supplement strategy to the current therapeutic methods for the treatment of RA. Recent research finds that herbal extracts and their purified compounds can selectively suppress the production of cPLA_2_ and eventually prevent the inflammatory process. Therein, a study demonstrates that *PG201* is an extract from a mixture contained 12 different herbs by ethanol. While *PG201* reduces the protein expression of cPLA_2_, it does not affect the mRNA expression level of cPLA_2_, which leads to the decreased production of PGE_2_, thus declining the concentrations of IL-1β, IL-6 and CC chemokine ligand-2 (CCL2) in supernatant and synovial tissues, eventually plays important anti-inflammation and anti-arthritic activity in LPS induced inflammatory cells (Raw264.7 cell) and RA rat model (Shin et al., 2003; Choi et al., 2012). All these effects mainly dependent on that *PG201* can substantially reduce the activator protein-1 (AP-1) and cyclic adenosine monophosphate-responsive element-binding (CREB) protein transcription factors’ DNA-binding activities, rather than nuclear factor-κB. Similarly, several fatty acids also can be hydrolyzed by sPLA_2_ at the position of sn-2 in the substrate phospholipid. However, the detailed mechanism of sPLA_2_ in the generation of eicosanoid is not clear in mammalian cells (Takada and Fujita, 2017). According to a study, the mice expressing sPLA_2_ enzyme (sPLA_2_ transgene) developed a deteriorated arthritis, which seemed more severe of arthritis than that of in mice lacking sPLA_2_. Since lipidomic analyses underlined the important roles of sPLA_2_ during the production of diverse PGs, especially PGI_2_, one possibility is that sPLA_2_ may aggravate the arthritis through increasing the levels of those eicosanoids. Meantime, the results of a primary study indicated that sPLA_2_ is induced production and activation in the process of inflammation and has been in a high level in the synovial fluid of RA patients (Dore and Boilard, 2019). So far, a number of studies focusing on this subject have no more accurate conclusions, and clinical trials of sPLA_2_ against RA cannot reach a satisfactory effect (Krizaj, 2014). But one study shows that the ethanol extract of *Boerhaavia diffusa*, a popular medicinal herb, concentration-dependently inhibits the sPLA_2_ enzymes to different degrees. Then, the inhibition of sPLA_2_ enzymes will neutralize the indirect hemolytic activity, mouse paw edema, and other RA symptoms induced by sPLA_2_ (Giresha et al., 2017). Also, both the Chinese herbal drug *Salvia miltiorrhiza* extract and the Indian synthetic herbal compounds 2-hydroxy-4-methoxy benzoic acid (HMBA) show a good inhibitory effect on sPLA_2_ induced toxicities (Gomes et al., 2012; Chen et al., 2017). Although results show good effects, some critical issues still exist and further investigations should to be done to verify the exact active ingredients. In view of the above findings, cPLA_2_ and sPLA_2_ are two valuable therapeutic targets, hence targeting the PLA_2_ enzyme mediator herbal products (such as *PG201*, *Boerhaavia diffusa*, *Salvia miltiorrhiza* extract and HMBA) development is likely to be a potential therapeutic strategy during the process of RA treatment.

### Cyclooxygenase (COX) and Prostaglandins (PGs)

Cyclooxygenase is an important enzyme that produces eicosanoids which regulate multiple pathological and physiological process. It converts AA into prostaglandin H2 (PGH2). Then, the PGH_2_ can be further catalyzed by different synthases to produce 5 main bioactive prostaglandins including PGD2, PGE2, PGF2, PGI2, and thromboxane A2 (TXA2) (Qureshi and Dua, 2020). Early studies have confirmed that there are two main subtypes of cyclooxygenase that are found in the human body (Nokhbehsaim et al., 2020). The first one is known as cyclooxygenase-1 (COX-1), which is considered as a beneficial enzyme and constitutively expressed in many kinds of tissues and cells, and acts as part of normal cellular housekeeping, such as maintaining the stomach’s lining, whereas the inducible enzyme named cyclooxygenase-2 (COX-2), in contrast, is induced by some particular conditions such as mitogenic and inflammatory stimuli, etc (Leng et al., 2018). Both types of COX make a class of compounds including prostaglandins, which produce signals that are short-lived and only affect nearby cells, or the same cells produce them. Long ago before regulation of COX-2 gene expression had been recorded in synovial tissues of both human and rodent. In the experimental arthritis, the expression level of COX-2 has been confirmed to increase in consistent with the clinical disease development and closely correlated with the infiltration of synovial mononuclear cell (Masferrer et al., 1994). In the human synovial tissues from RA, osteoarthritis (OA) and non-arthritic traumatic injury patients, the significantly express signal of COX was captured in a distinct structure called the synovial lining layer and multiple cell types including subsynovial synoviocytes, mononuclear inflammatory cells and vascular endothelial cells (Crofford, 1997). And the level of COX-2 was proved to be closely correlated with the infiltration degree of mononuclear cell, which provided a measurement manner for the synovial inflammation (Sano et al., 1992). During the occurrence and development of arthritis, the inflammatory cytokines such as IL-1β and TNF-α can induce the expression of COX-2 in synovial fibroblasts via stimulating the NF-κB and mitogen-activated protein kinase (MAPK) signals. Subsequently, COX-2 promoted the generation and accumulation of prostanoids in the synovium tissue (Nakano et al., 2020). Among the variety of prostaglandins, PGE2 and thromboxane A2 (TXA2) are the two powerful inflammation bioactive substances that contribute to the development of RA. In the course of RA, PGE2 causes the vasodilatation and subsequently brings about the neutrophils recruitment to the affected progression of joints (Peng et al., 2019). The neutrophils recruitment in joints is attributed to the generation of IL-17 induced by IL-23, as well as the damaged production of interferon-γ (IFN-γ) and IL-12. Furthermore, PGE2 regulates the degradation of matrix to influence the destruction of cartilage (Jiang L. et al., 2020). Under the inflammation condition, PGE2 can also induce the angiogenesis through accelerating the vascular endothelial growth factor (VEGF) production. Besides, research finds that PGE2 can not only promote the intension of inflammatory pain by also enhancing the sensitization of bradykinin and histamine-induced nociceptive stimuli, but also accelerate the plasma extravasation induced edema during the RA disease. In the meantime, studies have been shown that the effect of IL-1, IL-6 and TNF-α on bone resorption is mainly by a PGE2 dependent way (Jia et al., 2019). Extracts from a famous Chinese herbal called *Tripterygium wilfordii Hook. F* (TWHF) have been demonstrated effectively when treating the patients with inflaming and autoimmune diseases, for example, RA. In the subsequent experiments, researchers found that the chloroform/methanol extract (T2), ethyl acetate extract (EA) extract and the triptolide component could suppress the production of PGE2 via blocking the COX-2 upregulation in RASF of RA in a dose-dependent pattern (*p* < 0.05) in joint tissues of CIA mice (Tao et al., 1998; Lin et al., 2007). These alterations indicated that TWHF possesses promising immunosuppressive and anti-inflammatory activities on RA. In LPS stimulated neuroinflammation, treatment with *Myricetin*, a natural flavanol, can attenuate the expression of COX-2, which remarkably inhibits the generation of PGE2, IL-1β, and TNF-α, and eventually ameliorate the neuroinflammation (Jang et al., 2020). In human chondrocytes stimulated by IL-1β, *Myricetin* could increase the ration of p-Akt/Akt to activate the PI3K/Akt signaling pathway. Then, the activated pathway enhances the Nrf2/HO-1 pathway activation to abolish the NF-κB mediated inflammation, eventually suppresses the expression of COX-2 and inhibits the generation of inflammation mediators such as PEG2, IL-6 and TNF-α to ameliorate the development of OA (Pan et al., 2019). The above results supporting *Myricetin* can be regarded as a potential anti-RA herbal product by targeting the COX-2 and related PGE2 in the further research. Also, *Xanthones* extracts derived from *Swertia chirayita*, a famous Chinese herb, show a good anti-inflammatory property by inhibiting the expression of COX-2 and PGE2 in murine macrophage cells, which can be considered as a potential anti-RA herbal product for further study (Hu et al., 2019). Therefore, it is a beginning point for finding more products to decrease the level of PGE2 through suppressing the activity or expression of COX-2 during RA treatment. TXA2, the other product derived from COX, acts as a paracrine/autocrine hormone to induce the human platelets with as fast irreversible aggregation. It is also a potent smooth muscle contraction inducer by binding to its specific receptor, TXA2 receptor (Kashiwagi et al., 2019). Research shows that the biosynthesis of TXA2 at the molecular level in RA patients is obviously stronger than that of in healthy control subjects, suggesting its role in RA development. Further studies indicate that TXA2 binds to its receptor to active several intracellular signaling, which causes the transcription factor NF-κB activation, and subsequently increases the levels of IL-1 and TNF-α, leading to the synovial cell pathology in RA (Wang et al., 2015). Therefore, targeting TXA2 provides a potential therapeutic strategy during the RA treatment by herbal medicinal products.

### Lipoxygenase (LOX) and Leukotrienes (LT)

Lipoxygenase (LOX) is a kind of non-heme iron-containing dioxygenases that can catalyze the oxidation of fatty acid. Until now, the main subtypes of LOX, such as 5-LOX, 12-LOX, and 15-LOX, have been validated by several studies, which can stereospecifically combine with atom of oxygen at carbon atom 5, 12, or 15, respectively (Mackel et al., 2020). That much had been shown many times before: LOX transduction system is one major signaling pathways during inflammatory process of RA and that the synovial fluid of RA patients has a multitude of leukotrienes (Gheorghe et al., 2009). Studies show that 5-LOX and 15-LOX are primarily existing in the synovium of RA and OA, and therein 15-LOX is a lipid-per oxidizing enzyme that predominantly expressed in eosinophils, macrophages, fibroblasts and most articular tissues, including cartilage, synovium, and bone and its products are found in human synovial fluids, while 5-LOX is mainly located in the lining/sublining macrophages, neutrophils, and mast cells (Colamorea et al., 1999; Klein et al., 2004). In the human body, IL-13 induces the expression of 15-LOX in blood monocytes, while IL-4 stimulates the 15-LOX in RA synovial cells, monocytes, mast cells and dendritic cells. Then, 15-LOX converts AA to 15-hydroperoxy-eicosatetraenoic acid (15-HETE), which undergoes further conversion to form the15-hydroxyeicosatetraenoic acid (Wan et al., 2020). In the RA joint, the intermediate 15-HETE suppresses the generation of leukotriene B4 (LTB4) to regulate the leukocytes infiltration. Also, 15-HETE inhibits the mitogenesis of T-lymphocyte and the secretion of eosinophil leukotrienes C4 (LTC4), to prevent the neutrophil migration, and inhibit superoxide anion production and degranulation from activated neutrophil, which leads to the down-regulation of the inflammatory process in RA joints, indicating that 15-LOX has a protective effect on RA through forming the anti-inflammatory lipoxins (Wan et al., 2019). Some research shows that the expression of 15-LOX is elevated by IL-1β and TNF-α in RASF, while knockout of 15-LOX significantly reduces the cartilage destruction and inflammatory arthritis in C57/B6 mice induced by Freund’s Complete Adjuvant H37Ra (FCA; containing the 1 mg/ml *Mycobacterium tuberculosis* H37Ra) (Wu et al., 2012). Besides, treating with 15-(S)-HETE can also promote the osteoclasts differentiation (Kronke et al., 2009). The above studies indicate the indispensable role of 15-LOX during the arthritis pathogenesis, thus providing a valuable target for drug discovery and development when treating inflammatory arthritis. In RA, 12-LOX represents a similar anti-inflammatory enzyme operative, while the 5-LOX subtype catalyzes the synthesis of leukotriene B4 (LTB4) from arachidonic acid, and it is known to accelerate the pathogenesis of RA in contrast with 15-HETE. To confirm the role of 5-LOX in RA, RASF are pretreated with two kinds of 5-LOX inhibitors MK-886 (5 mM) and NDGA (5 and 10 mM) for 1 h. For the next 6 h, cells are treated with TNF-α (10 ng/ml). Researchers find that pretreatment of 5-LOX inhibitors can significantly decrease the TNF-α-induced IL-6 protein level and the expression of monocyte chemotactic protein-1 (MCP-1)/CCL-2, and the results are similar to those of 5-LOX knockdown (Lin et al., 2014). Furthermore, *in vivo* study demonstrates that 5-LOX inhibitor can alleviate the TNF-α induced phenotypic changes and even systemic inflammation. The alterations induced by 5-LOX inhibitors and 5-LOX knockdown suggest a valuable therapeutic strategy targeting 5-LOX for treating the RA (Chen et al., 2006).

Several studies show that many agents can inhibit the activity of LOX, which displays a significant inhibitory effect against joint inflammation. Among them, some herbal products have attracted people’s attention. *Bacopa monniera Linn* is described in the ayurvedic materia medica, as a therapeutically useful herb for the treatment of inflammation (Viji and Helen, 2008). In the carrageenan-induced rat paw edema model, *Bacopa monniera Linn* and its methanolic extract can significantly inhibit the activity of 5-LOX, while increasing the 15-LOX activity. Then, the decline in 5-LOX and elevation of 15-LOX activity leads to the decreased LTB_4_ production, which shows a significant effect in decreasing edema and the inflammation process. The result indicates that *Bacopa mon*niera *Linn* and its methanolic extracts may be a potential herbal product for treating joint inflammation and even RA when targeting LOX (+)-2-(1-hydroxyl-4-oxocyclohexyl) ethyl caffeate (HOEC) is an important herbal ingredient product isolated from *Incarvillea mairei var,* which has long been used as folk medicine for the treatment of inflammatory related diseases in China. Research found HOEC as an inhibitor of 5-LOX and 15-LOX *in vitro* to significantly inhibit both the two LOXs, thus suppressing the LOX related pathway in the beginning of arthritis. The inhibition of LOX pathway reduced the production of LTC4 and 15-HETE, but had little effect on LTB4 expression, eventually alleviating the clinical symptoms of arthritis, such as synovial hypeplasia, multiple cartilage destruction and pannus (Yang et al., 2018). These alterations suggest that treatment of herbal product HOEC exhibit a significant anti-RA effect by targeting the LOXs, which should be paid attention to and further studied. Also, a major herbal compound named *sec-O-glucosylhamaudol* (SOG), which is derived from *Saposhnikovia divaricata* (Turcz.) Schischk, has been reported to have anti-5-LOX activity, suggesting that SOG might have therapeutic effects on inflammatory disease, such as acute lung injury and RA (Zhao et al., 2016; Liu et al., 2020). Based on the above findings, targeting the lipoxygenase may be a great therapeutic strategy in treating inflammatory disease including RA and these herbal medicinal products will develop into effective medicines for RA treatment with the deepening and development of research.

### Matrix Metalloproteases (MMPs)/Tissue Inhibitors of Metalloproteinases (TIMPs)

Under the environment of RA, mononuclear/macrophages are activated to produce various cell factors and inflammatory mediators (Siouti and Andreakos, 2019). Among these mediators, the inflammatory factors such as IL-1β and TNF-α activate and accelerate the generation of matrix metalloproteases (MMPs), which will significantly increase the total activities of MMPs indirectly. The enzyme family can irreversibly promote the degradation of extracellular matrix (ECM) ingredients, including the collagen and fibronectin in the place of articular cartilage and bone (Viana et al., 2020). The major components of cartilage are type II collagen and proteoglycans, while type I collagen primarily constitutes the bone. Studies indicate that the process of MMPs mediated collagen degradation is the rate-limiting step during the damage of cartilage and bone. In the joints area, synovial cells produce the MMP-1, while MMP-13 synthesized by chondrocytes acid reside in cartilage (Li and Li, 2019; Nishi et al., 2019). Under the circumstances, MMP-13 degrades many substances including collagen, proteoglycan molecule and aggrecan. In a clinical study, researchers found the expression levels of MMP-1 and MMP-13 are increased in RA patients, and the baseline levels of serum MMP-1 and MMP-13 are correlated with disease progression, which can be used for predicting the radiographic and functional outcome in the early RA (Green et al., 2003). Regulated upon activation, normal T cell expressed and secreted (RANTES)/CCL5 is a chemokine produced by the majority of cell types, such as synovial fibroblasts, chondrocytes and activated T cells, etc. that participate in the pathogenesis of RA. In human RASFs, a study demonstrated that RANTES/CCL5 can induce the expression of MMP-1 and MMP-13, thus destroying the native collagen structure (Agere et al., 2017). Besides, RASFs produced MMP-1 and MMP-13, and the increased levels of these two MMPs in synovial fluid and tissue biopsies of RA patients offer some solid evidence for their function in tissue destruction (Yoshihara et al., 2000; Miller et al., 2009)**.** In the inflammatory arthritis mice model and SFs, Firestein et al. proposed that JNK signaling pathway can mediate the production of multiple cytokines such as IL-1β and TNF-α, and these cytokines subsequently induced the MMP-1 and MMP-13 expression (Han et al., 2001). In recent studies, researchers used JAK inhibitor or anti-TNF-α as intervention means and the results showed the obvious efficacy in ameliorating the tissue destruction, which was partly relation with the downregulation of MMP-1 and MMP-13 (Catrina et al., 2002; Boyle et al., 2015). The findings above indicated that MMP-1 and MMP-13 play a certain role in the RA progression, which should be considered as promising therapeutic target when treated with anti-RA agents, including herbal medicinal products. In the meantime, research also showed that the expression of other MMPs, such as MMP-2, MMP-3, MMP-9, MMP-12, and MMP-14, is obviously elevated in RA. And these enzymes degraded the components of non-collagenous protein of matrix, which results in the complete joint damage (Wang X. et al., 2019). Besides, MMPs also play a pivotal role in the angiogenesis progress, which is one of the critical components of the inflammatory arthritis pathogenic process (Withrow et al., 2016). Taken together, suppressing the activities of pathogenic MMPs in the section of joint, bone, and synovial cells can prevent or significantly decrease the joint and bone destruction, thereby alleviating the pain of arthritis patients and benefiting with an improved quality of life. Several studies report that TIMPs 1-4 are the natural inhibitors of MMPs, and they can diminish the pro-inflammatory cytokines and tissue damage in the joint (Hyc et al., 2016). Therefore, plenty of effort has been invested in finding and designing the effective suppressant and herbal products of MMPs’ activity and/or synthesis that display great anti-RA activity in several arthritis animal models. So far, there have been some herbal medicinal products that show good treatment results in treating the arthritis, especially RA. *Total glucosides of paeony* (TGP), a Chinese herbal product with extensive popularity, shows the obvious inflammation and pain inhibiting effects in a rat model of RA. Then, an in-depth study finds that part of its anti-RA effect is attributed to the suppression of the TNF-α and IL-1β production via macrophage-like synoviocyte (MLS), and that of MMP-1 and MMP-3 by FLS (Li et al., 2019). Moreover, this interdependent inhibition effect on different inflammatory enzymes and mediators can be explained by the fact that IL-1β and TNF-α regulates the expression and activity of MMP-1 and MMP-3 (Can et al., 2020). The anti-RA clinical application of *Pavlin* is well validated by the results acquired by above studies. Likewise, in a separate study, researchers find that *Triphala guggul*, an ayurvedic herbal medicinal product, shows a certain inhibition of key enzymes including hyaluronidase, collagenase and MMPs, which are involved in the tissue damage in RA (Sumantran et al., 2007). *Tanshinone IIA* (Tan IIA), the primary phytochemical extracted from a famous Chinese herbal Salvia Miltiorrhiza Bunge, is reported to be capable of promoting the RA-FLS apoptosis and inhibit arthritis progression in an AIA mouse model (Wang Z. et al., 2019). Moreover, RA patients treated at clinic with Tan IIA showed significant improvements in their clinical symptoms. After exploring, a researcher found that Tan *IIA* could effectively inhibit the increased mRNA expression of multiple matrix metalloproteinases (including MMP2, MMP3, MMP8, and MMP9) and proinflammatory factors in RA-FLSs stimulated by induced by TNF-α, resulting in the inhibition of inflammatory reactivity and end of knee joint destruction, which indicates the promising therapeutic role in the treatment of RA and shows potential to improve the life quality of RA patients (Du et al., 2020). Besides, Celastrol, Ikarisoside, and AKBA (mastic acid active ingredient) suppress the activity of MMP9, one of the transcription factor NF-κB-mediated genes (Choi et al., 2010; Xiao et al., 2018). Ursolic acid inhibits the expression of MMP-9, while the catechins extracted from *green tea* shows a good inhibitory effect on the expression and activity of MMP1 and MMP13 (Fechtner et al., 2017; Radhiga et al., 2019). *PG201*, the aforementioned new herbal drug, was reported to amplify the expression of TIMP-2, thus elevating the ration of TIMP2/MMP2 in synovial tissue and fluid in CIA rabbits and mice. For the arthritis progression, down-regulation of MMPs and up-regulation of TIMPs could prevent the collagen and proteoglycan release from cartilage to relieve the cartilage destruction and degradation (Park et al., 2005). Encouragingly, *PG201* (Layla, PMG Pharmaceutical) for treating OA had received the new drug application (NDA) approval in March 2012 (Yoo et al., 2014; Kim et al., 2016). Therefore, targeting the MMPs/TIMPs system may be a brilliant therapeutic strategy in treating the RA and these herbal medicinal products will develop into great medicines for RA treatment to a large degree.

### Nitric Oxide Synthase (NOS) and Nitric Oxide (NO)

Nitric oxide (NO) is a kind of radical gas with molecules synthesized from the guanidino group of l-arginine by nitric oxide synthase (NOS), which plays important roles in human body, including anti-tumor, anti-bacterial, wound healing, and vasodilation effects (Gartside et al., 2020). However, things always have two sides. When the level of endogenous NO is excess, severe inflammatory diseases such as inflammatory periodontal disease and joint disease including RA set in (Zaichko et al., 2020). NOS contain different isoforms: inducible macrophage type NOS (iNOS), endothelial cell NOS (ecNOS) and brain NOS (bNOS) (Gartside et al., 2020). Under the stimulation of a variety of immunological factors, pro-inflammatory cytokines can induce the non-hematopoietic cells, like fibroblasts, to produce the iNOS. Similar to that of NO, the production of iNOS may have either a toxic effect or a protective effect. Studies using non-selective NOS inhibitors showed the suppression of arthritis in rats, thus suggesting positive inhibitory effects of iNOS in acute and chronic joint inflammation, which indicate the possibility of direct NO toxic effects in RA (Stefanovic-Racic et al., 1994). In antigen-induced arthritis (AiA) mouse, Andreas et al. found that the development of AIA is related to the overexpression of iNOS in synovial microcirculation of knee joint, the elevated NO production and the increased leukocyte adhesion/infiltration in synovium (Veihelmann et al., 2001). Meantime, in the C57/Bl6 mice with AIA, the iNOS expresses, after stimulation, up to ten times more NO than the two constitutive forms of NOS (Schmitt-Sody et al., 2007). When detailed mechanisms are explored, researchers found that the activation of NF-κB in multiple arthritis models including RA, CIA and AIA is necessary for both the initiation and development of inflammation (Makarov, 2001). Overexpression of NF-κB has been reported to target the transcriptional process of iNOS mRNA, thus increasing the levels of iNOS gradually, which will catalyze and produce NO in arthritis models (Tak and Firestein, 2001). Under the inflammatory condition of RA patients, overexpression of iNOS is essential for the increased NO production (Aktan, 2004). At this point, researchers find that NO can stimulate the generation of pathogenic cytokines such as IL-1β, TNF-α, IFN-γ, and collagenase (Zaichko et al., 2020). Also, NO induces certain chemokines that contribute to the disease progression in RA. After decreasing the production of NO by inhibiting NOS, the arthritic symptoms were reduced. The above results demonstrate that suppressing the activity of iNOS could decrease the level of NO, eventually revealing an anti-arthritis effect, especially in RA, CIA and AIA. Based on these findings, the study of herbals shows that anti-oxidants extracted from a number of herbals can scavenge the NO and other free radicals. Besides, some herbals-derived compounds can suppress the activity of iNOS. *Daphne genkwa* is a herb with important anti-inflammatory effects. Some research showed that flavonoid is the main active ingredient. In LPS-induced RAW264.7 macrophages, T lymphocytes and fibroblast-like synoviocytes, *Daphne genkwa* can obviously inhibit the NF-κB pathway and down-regulate the expression of iNOS mRNA, which leads to the decreased expression of iNOS protein. After that, the decreased iNOS will reduce the secretion levels of NO and IL-6 in inflammatory RA-FLSs (Sun et al., 2020). According to one study, oral administration of POEa and POEe (ethyl acetate and ethyl ether extract of P. orientale, respectively) to rats can ameliorate the adjuvant arthritis (AA), and this is associated with decreased generation of various inflammatory mediators, including NO by macrophage (Gou et al., 2018). In another study, *Celastrus aculeatus* exerts its anti-inflammatory and anti-arthritis activity as tested in the AA model. After treating with *Celastrus* ethylacetate extract, rats show a significant decrease of the NOS expression and the NO levels both in serum and culture supernate of antigen-stimulated draining lymph node cells (Bai et al., 2014). Besides, *Celastrol*, as an ingredient of *Celastrus* and other celastraceae family of herbals, has been shown to modulate the expression of iNOS (Kannaiyan et al., 2011). Therefore, targeting the iNOS may be a potential therapeutic strategy in treating the RA and these herbal medicinal products will develop into great medicines for RA treatment.

### Indoleamine 2, 3-Dioxygenase (IDO)

Tryptophan is an essential amino acid that is critical for normal cell survival and proliferation, and obviously important for the development and functioning of many organs in the human body (Hu et al., 2020). Some studies found that tryptophan can be catabolized by indoleamine 2, 3-dioxygenase (IDO) to form the kynurenine, which can induce the apoptosis of T cells (Jiang X. et al., 2020). IDO, as the only one rate-limiting enzyme outside the liver that catalyzes tryptophan to kynurenine, is expressed in dendritic cells (DC) and activated macrophages, but not in the T cells. IDO positive DCs exert a significant role in the induction and maintenance of peripheral tolerance through the generation/activation of regulatory T cells (Treg) and consumption of self-reactive T cells. A study from Ozkan reports that the serum concentration of tryptophan markedly decreases, while the levels of kynurenine significantly increase in RA patients, indicating the integral role of IDO in RA disease (Merlo et al., 2017). Meanwhile, the concentration of kynurenine is higher in synovial fluid of RA than that of in OA patients and correlates with proinflammatory cytokines such as IL-1β, IL-6, and IL-8 expression (Bertazzo et al., 1999). Subsequently, IDO overexpression has been detected in the RA synovial, as well as in OA synovial. Under the inflamed synovium microenvironment, the expression of IDO was evaluated at a mRNA level in human FLS isolated from RA patients (Massalska et al., 2019). Some studies have found that IDO expression showed a direct effect on RA-related chondrocytes proliferation and collagen II in the matrix that suggests a possible effect on the MMPs (Chang et al., 2018). In the RA model, IDO could markedly diminish the accumulation of pathogenic Th1 and Th17 cells in the arthritic joints, thereby alleviating the severity of this disease. But with the deepening of the research, some results also show that inhibiting the activity of IDO may recede instead of aggravate RA. In-depth mechanism study finds that the IDO activity can be regulated by CD4^+^ CD25^+^ Treg and interferon-γ (IFN-γ). Moreover, the over-expression of enzyme tryptophanyl-tRNA-synthetase (TTS) in cytoplasmic can load the tryptophan into its specific tRNA, to form the chromoyl-tRNA complex, and this complex can antagonize the IDO-mediated deprivation of tryptophan, which will diminish the accumulation of T cells in arthritic joints and eventually recede the RA (Kim et al., 2018). Therefore, targeting the IDO could be a promising therapeutic strategy. So far, there has been no direct evidence that herbal medicine can alleviate the RA progression through targeting the IDO. However, in the depression-like model of rat, ethanol extracts from *Hemerocallis citrine* can attenuate the upregulation of proinflammatory cytokines and IDO (Liu et al., 2014). Meantime, *Feiji Recipe*, a compound of Chinese herbal medicine product, can significantly reduce the expression of IDO in C57BL/6 orthotopic mouse model (Luo et al., 2018). As of now, herbal products have not been studied much for their ability to modulate RA via altering IDO activity, but fortunately it also provides us with the great opportunity to apply the herbal products in the basic research and clinical treatment of RA in the future.

## Conclusion

Targeting the enzymes and their derivatives is likely to become a potential therapeutic strategy in the treatment of RA and these targeted herbal medicinal products would develop into great medicines for RA treatment. The major benefit of using these herbal products and their active ingredients due to their limited or almost no undesirable side effects. Hence, the interdisciplinary efforts of researchers are intended to explore the detailed biology functions of existing herbal products, to screen and identify novel herbal products, and to define the exact molecular mechanisms should be reinforced. These actions would facilitate and boost the discovery, screening, and development of safe and effective herbal products for treating RA and other inflammation, and autoimmune-mediated disorders.

**FIGURE 1:**
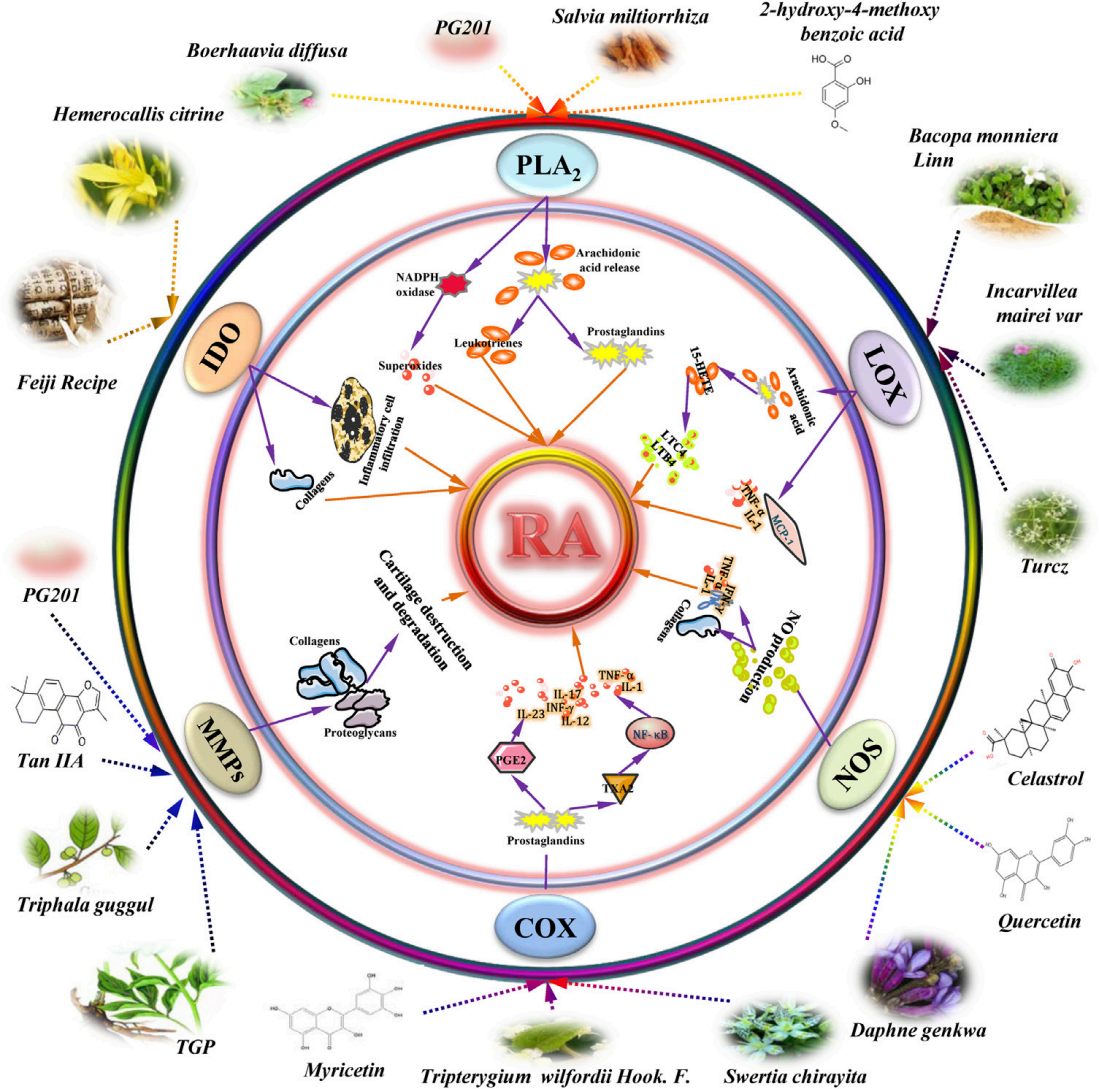
Schematic model representing the anti-rheumatoid arthritis (RA) effects of herbal medicinal products on therapeutic enzymes and their derivatives targets in RA. Additional abbreviations: 15-HETE: 15-hydroperoxy-eicosatetraenoic acid; COX-2: cyclooxygenase-2; PLA_2_: phospholipase A_2_; IDO: indoleamine 2, 3-dioxygenase; IFN-γ: interferon gamma; IL-1: interleukin-1; LTB4: leukotriene B4; LTC4: leukotrienes C4; LOX: lipoxygenase; MMP: matrix metalloproteinase; NOS: Nitric oxide synthase; NO: nitric oxide; PGE2: prostaglandin E2; Tan IIA: tanshinone IIA; TGP: total glycosides of paeony; TNF-α: tumor necrosis factor α; TXA2: thromboxane A2.

## Abbreviations:

COX-2, cyclooxygenase-2; cPLA2, cytosolic phospholipase A2; PG201, a multi-component phytopharmaceutical derived from 12 Oriental herbal medicines, Chaenomelis Fructus, Achyranthis Radix, Acanthopanax Cortex, Cinnamomi Cortex, Gentianae Macrophyllae Radix, Clematidis Radix, Angelica Gigantis Radix, Cnidii Rhizoma, Gastrodiae Rhizoma, Carthami Flos, Saposhnikoviae Radix, and Dipsaci Radix; HMBA, 2-hydroxy-4-methoxy benzoic acid; HOEC, (+)-2-(1-hydroxyl-4-oxocyclohexyl) ethyl caffeate; IDO, indoleamine 2, 3-dioxygenase; iNOS, inducible macrophage type NOS; LOX, lipoxygenase; MMP, matrix metalloproteases; NOS, nitric oxide synthase; OA, osteoarthritis; POEa/POEe, ethyl acetate and ethyl ether extract of P. orientale; RA, rheumatoid arthritis; SOG, sec-O-glucosylhamaudol; sPLA2, secreted phospholipase A2; TGP, total glucosides of paeony; TWHF, tripterygium wilfordii Hook. F.
